# The effect of group-based education on gastrointestinal symptoms and quality of life in patients with celiac disease: randomized controlled clinical trial

**DOI:** 10.1186/s12876-022-02096-1

**Published:** 2022-01-11

**Authors:** Zahra Akbari Namvar, Reza Mahdavi, Masood Shirmohammadi, Zeinab Nikniaz

**Affiliations:** 1 Tabriz University of Medical Sciences, Tabriz, Iran; 2 Nutrition Research Center, Tabriz University of Medical Sciences, Tabriz, Iran; 3 Liver and Gastrointestinal Diseases Research Center, Tabriz University of Medical Sciences, Tabriz, Iran

**Keywords:** Celiac disease, Gastrointestinal symptoms, Group-based education, Quality of life

## Abstract

**Background:**

In this trial, we investigated the effect of a group-based education program on gastrointestinal (GI) symptoms and quality of life (QOL) in patients with celiac disease (CD).

**Method:**

In the present study, 130 patients with CD who were on a GFD for at least 3 months, randomly assigned to receive group-based education (*n* = 66) or routine education in the celiac clinic (*n* = 64) for 3 months. We assessed gastrointestinal symptoms and quality of life using the gastrointestinal symptom rating scale (GSRS) questionnaire and SF-36 questionnaire at baseline and 3 months after interventions.

**Results:**

The mean age of the participants was 37.57 ± 9.59 years. There were no significant differences between the two groups regarding the baseline values. Results showed that the mean score of total GSRS score in the intervention group was significantly lower compared with the control group 3 months post-intervention (*p* = 0.04). Also, there was a significant difference in the mean score of SF-36 between the two groups 3 months post-intervention (*p* = 0.02).

**Conclusion:**

Results showed that group-based education was an effective intervention in patients with celiac disease to improve gastrointestinal symptoms and quality of life.

*Trial registration* IRCT code: IRCT20080904001197N21; registration date: 5/23/2019.

## Background

Celiac disease (CD) is a chronic inflammatory condition of the small intestine that is triggered by the ingestion of gluten [1]. The global prevalence of the CD is about 1% and its prevalence in Iran is similar to global reports [2]. CD is associated with different gastrointestinal and extra-gastrointestinal symptoms including dermatitis, headache, foggy mind, fatigue, joint problems, anemia, poor growth, and infertility [3–5].

Complete elimination of gluten from the diet is the only available treatment [6, 7]. Non- adherence to a gluten-free diet (GFD) leads to a reduction in quality of life and worsening of symptoms [8–10]. However, strict compliance to this diet can be difficult [11, 12]. A recent study in Iran indicated that 51.2% of Iranian patients with CD had higher than normal levels of serum anti-tTG-IgA [15].

Considering the widespread distribution of gluten in different food, drugs, and commercial products, a complete education program should be implemented to increase the patients’ knowledge about the gluten-free diet. Individual education provided by an expert dietitian is usual care of these patients. However, this method is time-consuming and despite this training, studies have shown that the adherence of patients to GFD is low [13, 14]. So, different studies have focused on evaluating various methods of nutrition education such as online education, education through the smartphone application, and education by text messages on patients’ symptoms and quality of life [16–18].

Group-based education is another method of nutrition education. This method provides more detailed information and support from other patients who experience the same condition and promotes discussions about patients' problems [19, 20]. Different studies have assessed the effect of this method in different conditions and reported its promising effect in diabetes and gastrointestinal disease [21–23]. Two studies also investigated the effect of this method in patients with celiac disease. Jacobbson et al. reported that celiac school improves psychological health and gastrointestinal (GI) symptoms in patients with CD [24]. Rej et al. also reported that in patients with newly diagnosed CD, group clinics had a positive effect on patients' QOL [25].

Considering that only a limited number of studies had investigated the influence of group-based education on celiac patients, we designed a randomized controlled clinical trial (RCT) to compare the effect of group-based education and individualized education programs in patients with CD. we reported the result of the programs on knowledge and adherence levels in our previous report [26]. In the present report, we provided the result of the effect of the group-based education program on gastrointestinal symptoms and quality of life in patients with CD.

## Methods and material

In this, parallel designed RCT, celiac patients were selected from the East-Azerbaijan celiac registry database. The detail of patient selection was reported in our previous report [26]. As yet, 450 patients were registered in East-Azerbaijan registry database, and the patients selected for inclusion in the present study by random sampling according to the inclusion criteria. The biopsy-proven patients with ages ranged between 18 and 55 years were involved. Moreover, for inclusion in the present study, the patients should have the reading and writing ability and were on a GFD for at least 3 months. Also, we excluded patients with self-reported concomitant diabetes. All included were educated individually on a GFD through pamphlets.

The protocol of this study was accepted by the ethics committee of Tabriz University of Medical Sciences (IR.TBZMED.REC.1398.075) and all methods were performed in accordance with declaration of Helsinki. Patients signed written informed consent before participation.

This trial was registered in the Iranian registry of a clinical trial (IRCT) [registration number: IRCT20080904001197N21; registration date: 5/23/2019] and follows the CONSORT guidelines [27].

### Sample size calculation

The sample size for the present study was calculated by G-power considering the result of a previous study [16] and the power of 80% and the confidence interval of 95% that necessitate a total sample size of 120 patients. Considering the dropout rate of 18%, 140 patients were recruited.

### Interventions

A computer generation randomization list was used for the random allocation of 140 patients to two groups. This procedure was accomplished by an independent person who did not participate in the other processes of this trial. The randomization list was concealed using sequentially numbered, opaque, sealed envelopes.

Sixty-six patients in the intervention group attended an education class of 8–10 patients. An expert dietitian conducted all educational sessions in the intervention and the control group with the same educational content. This program included three sessions lasting approximately 1 h each session with discussing the following topics: (1) Celiac disease etiology, diagnosis, and treatment, (2) widespread information about gluten and GFD, and 3() interpretation of commercial product labels. Lectures, interactive learning/skills training, and group discussion were used for transmitting the information.

In the control group, the patients continued the three individual-based education sessions in the CD clinic with the same topic provided in the group-based education sessions. Every meeting of individual-based education lasted 45–60 min.

### Outcome measure

Assessing the gastrointestinal symptoms and quality of life were the primary outcome of the present trial.

Gastrointestinal Symptom Rating Scale score (GSRS) questionnaire was used to assess the GI.

Symptoms. GSRS includes 15 questions that have response options ranging from “no symptoms (0)” to “most pronounced symptoms (6)”. The questionnaire includes five sections that asking about diarrhea, constipation, abdominal pain (each includes three questions), reflux (two questions), and indigestion (four questions). The questionnaire was translated into the Persian language and validated in our population previously [28]. GSRS was completed before the initiation of the study and 3 months post-intervention.

For evaluating the QOL, the SF-36 questionnaire was used. This questionnaire includes 36 questions yielding two summary measures: physical component score (PCS), and mental component score (MCS). The PCS includes four scales of physical functioning, physical role limitation, bodily pain, and general health. The MCS is composed of vitality, social function, emotional role limitation, and mental health. For each domain, a score ranging from 0 to 100 with a higher score indicating better health. The questionnaire was completed before the interventions and 3 months post-intervention.

#### A blind researcher for randomization scored the questionnaires

The participants were asked not to get any information from other sources and contact researchers if they had any concerns.

### Statistical analysis

SPSS 16.0 was used for data analysis intention-to-treat analysis (ITT) approach was applied. Kolmogorov–Smirnov test was used for assessing the normal distribution. The mean and standard deviation (SD) was computed for the continuous variable and frequencies and percentages were calculated for nominal and ordinal variables. For analysis of the changes within each group, we applied a paired sample t-test for normally distributed data and Wilcoxon signed-rank test for not normally distributed data. Independent sample t-test was used for the analysis of the between-group comparisons. A Chi-square test was applied for analysis of the nominal and categorical variables. One-way analysis of covariance (ANCOVA) and rank ANCOVA was used to examine the changes in post-intervention values by adjusting to baseline values and confounding factors including age, sex, and presence of celiac symptoms, co-morbidities, and level of education. The significance level was chosen at 5%.

## Results

**Fig. 1:**
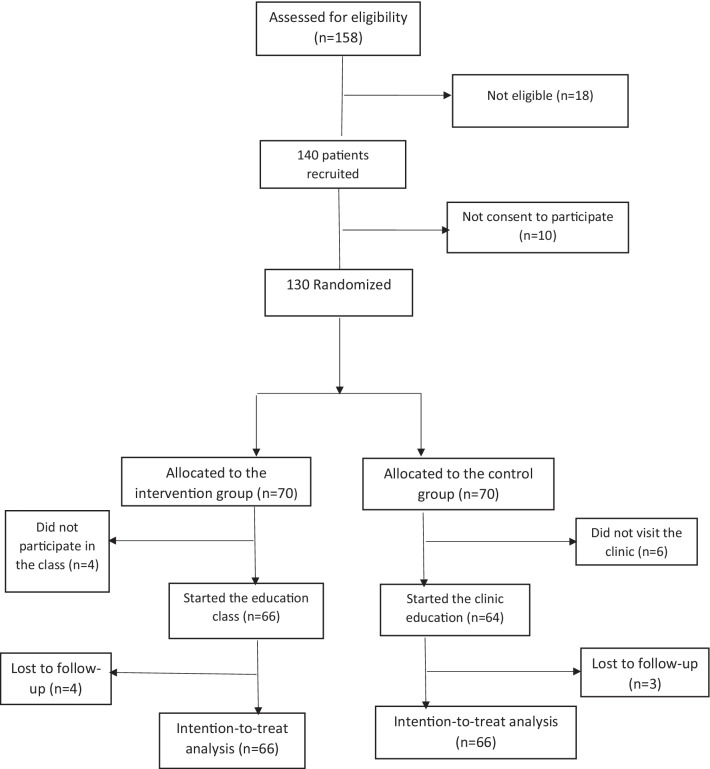
Flow chart of patients’ recruitment and analysis

In this trial, 10 patients refused to attend the classes, so the study continued with 130 patients (66 in the intervention group, and 64 in the control group). The detail of participant attendance in the study was provided in Fig. 1. Briefly, four patients in the intervention group were lost two follow up and in the control group, four patients did not finish the follow-up (three due to a loss to follow-up and one due to death (because of unrelated causes).

As illustrated in Table 1, the baseline mean (SD) age of the patients was 37.4 (9.86) years in the intervention group and 37.7 (9.39) years in the control group. The patients followed a gluten-free diet for a mean (SD) of 4.78 (3.40) years. The baseline characteristics of the participants in the two groups were not significantly different.

**Table 1:** The baseline characteristics of participants

Variable	Intervention group (*n* = 66)	Control group (*n* = 64)	*p* value
Age (years)	37.40 ± 9.86	37.75 ± 9.39	0.84*
*Sex, n (%)*			0.43**
Male	26 (39.4)	21 (32.8)	
Female	40 (60.6)	43 (67.2)	
*Educational level, n (%)*			0.46**
≤ Diploma	40 (60.6)	37 (57.8)	
College	26 (39.4)	27 (42.2)	
*Marital status, n (%)*			0.83**
Single	1(25.8)	14 (21.9)	
Married	49(74.2)	5 (78.1)	
Following gluten-free diet (year)	4.78 ± 3.40	4.25 (3.31)	0.37*
Family history of CD, *n* (%)	5 (7.6)	5 (7.8)	0.94**
Presence of comorbidities, *n* (%)	13(19.7)	20 (31.3)	0.13**
GSRS total score	2.01 ± 1.29	1.91 ± 1.44	0.66*
MCS	52.55 ± 19.54	56.51 ± 24.19	0.30*
PCS	57.46 ± 61.47	60.21 ± 24.40	0.50*
Total QOL	55.00 ± 19.46	58.36 ± 23.17	0.34*

*CD* celiac disease, *GSRS* Gastrointestinal Symptoms Rating Scale, *QOL* quality of Life; *MCS* Mental Component Score, *PCS* Physical Component Score

^*^p value of between group comparison

The comparison of the changes in GSRS total score and subscores between the two groups is shown in Table 2. In both groups, the mean post-intervention GSRS total score was not significantly different from the baseline score. However, 3 months post-intervention, the mean total score of GSRS was significantly lower in the intervention group compared with the control group (*p* = 0.04) after adjusting for baseline values. Considering GSRS different domains, the mean abdominal pain (*p* = 0.04) was significantly lower in the intervention group in comparison with the placebo group three-month post-intervention.

**Table 2:** Within-group and between-group comparison of the mean GSRS total score and subscores

GSRS clinical syndromes	Intervention group (*n* = 66)		*P* value*	Control group (*n* = 64)		*P* value*	*P* value**
	Baseline	Post intervention		Baseline	Post intervention		
Abdominal pain	2.30 ± 1.92	1.86 ± 1.82	0.04	1.86 ± 1.36	2.15 ± 1.84	0.16	0.04
Reflux^¥^	1.50 ± 1.74	1.36 ± 1.81	0.39	1.59 ± 2.11	1.68 ± 1.80	0.65	0.29
Diarrhea^¥^	1.71 ± 1.83	1.48 ± 1.60	0.20	1.81 ± 2.03	1.99 ± 2.25	0.38	0.06
Constipation	2.36 ± 2.10	2.22 ± 1.95	0.56	2.13 ± 2.14	2.27 ± 2.00	0.47	0.51
Indigestion	2.19 ± 1.70	2.15 ± 1.63	0.81	2.18 ± 1.80	2.27 ± 1.87	0.61	0.63
Total score	2.01 ± 1.29	1.82 ± 1.24	0.11	1.91 ± 1.44	2.07 ± 1.52	0.25	**0.04**

*GSRS* Gastrointestinal Symptoms Rating Scale

^*^Before-after analysis; **Between group analysis; ^¥^non normally distributed

**Fig. 2:**
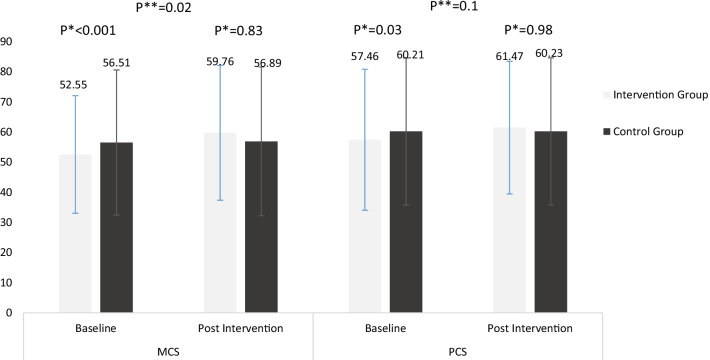
Within group and between-group comparison of mean quality of life scores. *PCS* Physical Component Score, *MSC* Mental Component Score. *p value of within group comparison; **p value of between-group comparison

A comparison of the QOL score between the two groups is shown in Fig. 2. The mean post-score of MCS (*p* < 0.001) and PCS (*p* = 0.03) of SF-36 were increased significantly in the intervention group. No significant changes were observed in the control group. The results of the ANCOVA test showed that the mean post-intervention MCS of SF-36 in the intervention group was significantly higher compared with the control group (*p* = 0.02).

## Discussion

Compliance with a gluten-free diet (GFD) is the only available treatment for CD and adequate knowledge about CD and GFD had been shown to have an important role in patients' adherence to the diet [26]. In this trial, we assessed the effect of group-based education on gastrointestinal symptoms and quality of life in adult patients with CD. The superiority of group-based interventions over individual-based interventions on gastrointestinal (GI) symptoms in different gastrointestinal diseases has been shown. Ringstrom et al. showed that group-based education had a greater impact on GI symptoms and GI-specific anxiety compared with written information in IBS patients [22]. Urnes et al. showed improved quality of life and digestive symptoms in patient with reflux disease who participated in a group education program [29]. In accordance with these results, we found that the post-intervention mean score of GSRS, in the intervention group was significantly lower than those in the control group. To the best of our knowledge, only one study had reported the effect of group-based education on GSRS score in patients with CD and showed that abdominal pain score was significantly improved in the intervention group compared with the control group ten weeks after intervention. [24].

The superiority of group-based education in improving patients' GI symptoms may be related to the fact that this method of education has a positive effect on GI symptoms through increasing the knowledge of patients about CD and GFD and also improving dietary adherence. In the previous study, we identified that group-based education in adults with CD had a significant effect on the score of adherence to the GFD, compared with the usual individual education [26]. The gut can influence the blood–brain barrier through secretion of gastro-intestinal-derived hormones, small molecules, and production of metabolic cofactors that lead to production of inflammatory components. This systemic inflammation in CD is associated with depression, and psychiatric comorbidities [30, 31]. So, by improving patients` adherence, we can expected an improvement in symptoms. In some patients with other gastrointestinal disorders, reducing stress, anxiety or depression, can lead to a reduction in gastrointestinal symptoms, and group training by improving psychological quality of life has a positive effect on gastrointestinal symptoms [22, 29]. Clinical improvement in some psychiatric symptoms such as depression, anxiety, and irritability was observed after administration of a GFD in CD [32]. This may partly justifiable by this theory that the patients may worried about their symptoms and after having a diagnosis and resolution of symptoms, also depression and anxiety alleviate. However, some contradictory results were also reported in previous studies where these symptoms have even increased after beginning of GFD [33]. These observations may be due to this fact that psychiatric symptoms and quality of life are complex issues.

The result of the present trial indicated a higher score of QOL in the intervention group compared with the control group. Jacobsson et al. also reported the higher QOL score in the group-based education group in comparison with individual-based education in women with CD [34]. Group-based education comfort discussions and provides more detailed information and patients found the information given during a group session, easier to understand. Moreover, studies indicated that asking questions and discussing them together with other patients and the instructor, resulted in more assurance [19, 22]. Also, in the group-based education program, the patients with the same concerns share and reveal their thoughts, and feelings and find better solutions to their problems [20]. Moreover, in the group-based education, the patients find more other patients with similar concerns, and their anxiety level about the disease was decreased and they become more motivated to increase their adherence to GFD, and consequently their quality of life improve [29, 35, 36]. However, Rej et al., showed that group-based education of newly diagnosed patients with CD has no significant effect on QOL [25]. The discrepancy between the results of the present study and Rej et al. study partly can be attributed to the characteristics of included participants since they included the newly diagnosed patients.

This trial had some limitations as follows. Considering the inclusion criteria of the present study about the age group and education status of participants, the results may have limited generalizability. Moreover, for assessing the quality of life of patients, we did not use the disease-specific questionnaire. However, different studies used the SF_36 questionnaire for assessing the quality of life in celiac disease. Also, the duration of follow-up was limited. In addition, we did not obtain the patients' subjective feedback about this method of education.

## Conclusion

The results of the present study showed that group-based education had statistically significant effects on improving GI symptoms as well as QOL compared with routine education. Thus, in term of celiac disease, in addition to the individual education program, a group-based education program should also conduct to increase the patients’ adherence to GFD and consequently improve their symptoms and increase their QOL. Considering the limitations of the study, more studies with longer durations of follow-up and using disease-specific questionnaires, and assessing the patients' subjective feedback about the method of education are needed to prove these results [37].

## Data Availability

The dataset supporting the conclusion of this article is included in the article. The data will be available by reasonable request made to corresponding author, Zeinab Nikniaz.

## Abbreviations

CD: Celiac disease
GFD: Gluten-free diet
ITT: Intention to treat
GSRS: Gastrointestinal symptom rating scale
QOL: Quality of life
ANCOVA: One-way analysis of covariance
